# Recent Insights into the Population Genetics and Dynamics of the Inherited Disorders of Hemoglobin

**DOI:** 10.4084/MJHID.2009.022

**Published:** 2009-12-20

**Authors:** D.J. Weatherall

**Affiliations:** Weatherall Institute of Molecular Medicine, John Radcliffe Hospital, Headington, Oxford, UK

## Abstract

The inherited disorders of hemoglobin are by far the commonest monogenic diseases and there is considerable evidence that they have reached their very high frequencies due to heterozygote advantage against malaria. Recent studies have begun to clarify the effect of interactions between malaria and some of the more severe inherited hemoglobin disorders and demonstrated how complex epistatic interactions between different hemoglobin variants with respect to malaria resistance and modification of their phenotypic severity may explain the remarkable heterogeneity of distribution and the frequency of these conditions both between and within individual populations.

## Introduction:

It was estimated recently that over seven million babies are born each year with either a congenital abnormality or genetic disease, and that up to 90% of these births occur in low or middle-income countries.^1^ Of these conditions, approximately 25% are made up of five disorders, two of which, the inherited disorders of hemoglobin and glucose-6-phosphate dehydrogenase deficiency, are monogenic diseases. It is estimated that in excess of three hundred thousand children are born each year with either sickle cell anemia or one of its variants or one or other form of thalassemia.

There is now extensive evidence that the extremely high frequency of the hemoglobin disorders compared with other monogenic diseases reflects natural selection mediated by the relative persistence of carriers against *P. falciparum* malaria. Other factors that maybe involved include the widespread practice of consanguineous marriage, increased maternal age in the poorer countries, and gene drift and founder effects.

Over recent years there has been a major revival in interest in the study of interactions between the inherited hemoglobin disorders and *P. falciparum* malaria, work that has been the subject of several extensive reviews.^2^,^3^ Here, we shall focus on recent developments in this field, particularly with respect to interactions between malaria and more severe forms of the hemoglobin disorders and how a study of this interplay is starting to provide some insights into the remarkable variability of the distribution of these diseases among different populations. We shall also address briefly some of the potential practical implications of this new information.

## The current state of malaria transmission:

Despite progress towards its control malaria is still the most important parasitic disease. It is estimated that some three billion people reside in malarious areas and that the disease is responsible for between one and three million deaths each year. Until recently it was thought that only four species of malarial parasite (*Plasmodium*) have humans as their natural hosts; *P. falciparum, P. vivax, P. malariae,* and *P. ovale*. More recently it has been found that many cases of malaria that were previously diagnosed as being due to *P. malariae* infection are in fact due to a fifth parasite, *P. knowlesi*, particularly in Malaysia and Borneo.^4^

Until recently, it was thought that the *P. falciparum* malaria is by far the most severe form of the condition and, globally, the major cause of mortality. However, over recent years there has been increasing evidence that malaria due to *P. vivax*, which occurs at extremely high frequencies in parts of Asia and South America, may be a much more serious condition than was previously realised.^5^,^6^ Recent evidence suggests that it may cause many of the serious complications of malaria that have usually been associated only with *P. falciparum*, involvement of the brain for example. Thus in considering the interactions of malaria with the haemoglobin disorders it is important now to focus not only on *P. falciparum* but also on *P. vivax* malaria.

## Malaria and structural hemoglobin variants:

There is now extensive evidence suggesting that relative resistance on the part of heterozygotes to *P. falciparum* malaria has been the major factor underlying the extremely high gene frequencies of hemoglobins S, C and E.

### Hemoglobin S

Early comparative studies of parasite rates and density in children with the sickle cell trait and controls, together with the discovery of the relative rarity of Hb S in patients with severe malaria in Africa, provided convincing evidence that the sickle cell trait provides at least some degree of protection against severe malarial infection.^7^ More recent studies in East Africa suggest that the major impact of Hb S seems to be on protection against either death or severe disease, that is profound anemia or cerebral malaria, while having less effect on infection rates *per se*.^8^ Indeed, these studies indicate that Hb S carriers have approximately 60–80% protection against the severe complications of malaria. Several cellular mechanisms that might underlie this protective effect have been clearly defined^3^ although there are also data which suggest that an immune mechanism may also be involved.^9^

For many years it was believed that at least part of the extremely high mortality of babies with sickle cell anemia in Africa might reflect death due to malarial infection. However, in a recent study carried out on the coast of Kenya no evidence was found to support the concept that the risk of malaria is higher among children with sickle cell disease than among normal children.^10^ In fact, a related study from the same region has demonstrated that the pattern of infection in the early years of life of children with sickle cell disease is very similar to that in Western countries.^11^ If confirmed in other regions, these findings have important implications regarding antibiotic and malaria prophylaxis for patients with this disease in malaria-endemic regions.

### Hemoglobin C

Work in West Africa has demonstrated quite unequivocally that the relatively high frequencies of Hb C have also been maintained by resistance to *P. falciparum* malaria 12. In this case it appears that both heterozygotes and homozygotes are protected, suggesting that Hb C could be an example of a transient polymorphism, that is a variant that might be moving to fixation in a population in which the selective process continues. This concept is based on the apparent lack of clinical disability in Hb C homozygotes. However, further work is required to assess whether homozygotes for Hb C are, in genetic terms, completely fit, that is whether the homozygous state is associated with absolutely no clinical disability.

Recent studies suggest that the expression of the malarial antigen PfEMP1, an important adhesion molecule, is reduced in Hb C-containing red cells, an effect that is most marked in homozygotes.^13^ The functional importance of this finding compared with the unusual rheological properties of Hb C-containing red cells remain to be determined.

### Hemoglobin E

Hb E, which produces a mild thalassaemia phenotype in homozygotes, occurs at an extremely high frequency in parts of the Indian subcontinent, Bangladesh, Myanmar, and throughout Southeast Asia, reaching carrier rates of up to 70% in parts of northern Thailand and Cambodia. So far there have been no formal case control studies to analyse the interaction between Hb E and malaria but population studies in Thailand have suggested that those with the Hb E trait show less severe disease when admitted to hospital with acute *P. falciparum* malaria.^14^ Furthermore, convincing *in vitro* culture studies have shown that the red cells from Hb E heterozygotes, but not homozygotes, are more resistant to invasion by *P. falciparum*.^15^

## Thalassemia and *P. falciparum* malaria:

Although the malaria hypothesis, as first proposed by Haldane, was first developed to explain the high frequency of the thalassemias it has taken many years to confirm that Haldane was correct. There is now extensive evidence to suggest that the mild forms of α thalassemia reach their extraordinary high frequencies due to protection against *P. falciparum* and at least some suggestive evidence that the same applies to the β thalassemias.

### α^+^ thalassemia:

The α^+^ thalassemias, which are most commonly due to deletions of one of the linked α globin genes, −α/αα, are the commonest monogenic diseases in the world population. They occur in a broad band stretching through sub-Saharan Africa and the Mediterranean, through the Middle East and the Indian subcontinent to Southeast Asia. The frequency in these regions varies fro 5–40% although in parts of Northern India and Papua New Guinea close on 80% of the population are carriers.^16^

Extensive population studies in the Southwest Pacific showed a strong correlation between the distribution of α^+^ thalassemia and malaria, and related molecular analyses indicated that the particular form of α^+^ thalassemia in these populations is different to that and the Asian mainland.^17^ An unusual feature of the distribution of α^+^ thalassemia in this region is that it is also found in Fiji in the west and Tahiti in the east and in other populations in which malaria has never been recorded. However in these regions all the α^+^ thalassemias can be accounted for by a single mutation which was first identified in Vanuatu, suggesting that their occurrence in these areas has been the result of population migration and founder effect.^18^

These findings were later augmented by case control studies in Papua New Guinea where it was found that, compared with normal children, the risk of being admitted to hospital with severe malaria was significantly reduced, both for α^+^ thalassemia homozygotes and heterozygotes.^19^ More recently these findings have been replicated in several African populations and it is now absolutely clear that the α^+^ thalassemias do offer protection against *P. falciparum*.^20^,^21^ Both cellular and immune mechanisms have been found that may offer at least a partial explanation to the protective effect of α^+^ thalassemia against malaria. The red cells of individuals with α^+^ thalassemia bind more malaria-immune globulin than normal and appear to be more susceptible to phagocytosis *in vitro*.^22^ In particular, they are less able than normal to form rosettes, an *in vitro* phenomena whereby uninfected red cells bind to infected cells. This may affect a reduced expression of complement receptor 1 (CR1) expression on α thalassemic red cells.^23^ These cells are also less able to adhere to human umbilical vein endothelial cells.^24^ Taken together it does appear that α thalassemic red cells may be less able to sequester in blood vessels, an important mechanism for virulence of infection. It has also been suggested recently that the relatively high red cell counts in this condition may offer some degree of protection against the profound anemia of malaria in early life.^25^ We will consider other possible immune mechanisms mediated by α^+^ thalassemia in a later section.

### β thalassemia:

With the exception of a small-scale case control study in Liberia, which suggested that the β thalassemia trait is protective against severe malaria, there have been no large-scale studies of this type.^26^ There is however a considerable body of epidemiological data relating the frequency of β thalassemia to malaria transmission that indicates that it too may be a protective polymorphism against malaria, a conclusion that is strengthened by the pattern of haplotype analysis of the β globin genes in relationship to different β thalassemia mutations.^16^ Furthermore, work in both human blood cells^27^ and in the cells of transgenic mice carrying human γ globin genes,^28^ it has been found that red cells which contain human fetal hemoglobin are associated with ineffective development of *P. falciparum* or *P. yoelli*. Since there is clear evidence that the rate of decline of fetal hemoglobin production after birth is delayed in β thalassemia heterozygotes^16^ this could offer a further mechanism of protection during the first year of life.

## *Plasmodium vivax* infection and the hemoglobinopathies:

Early studies in Africa,^29^ and later in Papua New Guinea,^30^ revealed a very high frequency of the Duffy-negative blood group phenotype in populations in which malaria is common. Red cells lacking the Duffy determinant are resistant to invasion by both *P. vivax* and *P. knowlesi*, explaining the almost complete absence of *P. vivax* malaria in many parts of Africa.^31^ This red cell phenotype results from a mutation in a GATA-1 binding site in the gene for the Duffy antigen chemokine receptor (DARC) which prevents its expression and hence the Duffy negative phenotype.^32^

Given this remarkable example of a malaria protective polymorphism it is surprising that there have been, to date, very few studies of the possible interaction between the hemoglobin disorders and *P. vivax* malaria. In a cohort study of babies in Vanuatu it was found, surprisingly, that those with α thalassemia had a higher frequency of malaria during the very early years of life than normal infants and that this was most marked in the case of *P. vivax* malaria.^33^ This led to the suggestion that one of the possible mechanisms of resistance to falciparum malaria in children with α thalassemia might reflect early immunization with *P. vivax* malaria, the later protection against *P. falciparum* reflecting cross-immunization between the two varieties of malarial parasite.^33^ A later study in Papua New Guinea also observed a highly significant increase in parasitemias in babies with α thalassemia compared with normal infants.^19^ There is a possible mechanism for this observation. The Duffy antigen is expressed at a greater level in young red cell precursors and hence in any condition where there is a rapid turnover of red cells, and hence a younger red cell population in the peripheral blood, an increased susceptibility to *P. vivax* might occur.

Very recently a study has been reported from Sri Lanka in which the susceptibility of children with HbE β thalassemia to both *P. vivax* and *P. falciparum* malaria was assessed.^34^ Compared with control subjects, it appeared that those with HbE β thalassemia were more susceptible to both forms of malaria, but particularly to *P. vivax*. Interestingly, those who had been splenectomized appeared to be particularly susceptible, but even the malarial-antibody levels in children with intact spleens were significantly higher than those in age-matched controls. Evidence of previous vivax infection was also related to spleen size and hence to the severity of the thalassemic phenotype. Again, it is possible that the increased susceptibility to *P. vivax* reflects the rapidly-turning-over red cell population of children with HbE β thalassemia.

Clearly therefore it will now be important to study the effects of the heterozygous states for β thalassemia and hemoglobin E with respect to susceptibility to *P. vivax*. From a clinical point of view, since *P. vivax* malaria is presenting an increasingly serious health problem in Asian populations where HbE β thalassemia is so common it will also be important to repeat these studies in other parts of Asia to determine whether *P. vivax* prophylaxis should become an integral part of thalassemia control programs in countries where this form of malaria is particularly common.

The significance of interactions between different malaria-resistant polymorphisms

Since different hemoglobin variants or forms of thalassemia that offer protection against malaria frequently occur together in the same population it is important to determine how they interact with one another with respect to the level of malaria protection. The term epistasis is used to describe potential interaction between two or more loci of this kind. Interestingly, the first study of this kind, in east Africa, has shown that there is negative epistasis between the sickle cell and α thalassemia genes with respect to protection against *P. falciparum*.^35^ As discussed above, while the sickle cell trait or the heterozygous or homozygous state for α thalassemia alone provide significant protection, this effect is almost completely nullified in those who are heterozygous for Hb S and homozygous or heterozygous for α^+^ thalassemia. This may reflect the intracellular level of Hb S. Those who inherit the α thalassemia gene together with the sickle cell trait have significantly lower levels of Hb S compared with those who have the sickle cell trait alone.

Recently, there has been a particularly interesting twist to the story of these interactions with respect to the distribution of the sickle cell and thalassemia genes in Africa and the Mediterranean populations. Although the sickle cell gene occurs to a modest degree in some though not all Mediterranean populations the predominant hemoglobin variants in these regions are the α and β thalassemias. On the other hand, β thalassemia, with the exception of some localized parts of Liberia, is relatively rare across the African subcontinent, where the sickle-cell and α thalassemia mutations predominate. It has been shown recently that this state of affairs can be explained by two epistatic interactions, one that we have just described in the case of the sickle cell trait and α thalassemia, together with the ameliorating effect of α thalassemia on the phenotype of the severe forms of β thalassemia.^36^ By modelling these two interactions within populations it has been possible to explain much of the present discrepancies in the regional distribution of these hemoglobin variants in Africa and the Mediterranean region. Since the disparate distribution of different hemoglobin variants in different parts of the world has always remained a puzzle, it will be interesting to apply a similar approach to their distribution in other populations.

## Conclusion:

In this short review I have tried to highlight some recent information obtained from the studies of the relationship between the hemoglobin disorders and malaria, set against the background of the considerable amount of progress that has been made in this field over recent years. Several aspects of this work have interesting and potentially important implications for the hemoglobin field.

Currently, we have only an extremely approximate estimate of the global burden of disease that is being produced by the hemoglobin disorders. This is largely due to lack of accurate information about gene frequencies in individual populations 16. Recent micro-mapping studies suggest that, even within relatively small population groups, Sri Lanka for example, there is a remarkable heterogeneity in the distribution of the hemoglobin variants.^37^ While it always seemed possible that this reflected similar heterogeneity in the distribution of malaria, and indeed this appears to be the case in Sri Lanka, it is now apparent that it may also result from complex epistatic interactions, not just between the variants themselves but also with respect to the resulting effect on malaria resistance. The epistatic interaction between the sickle cell and α thalassemia traits in Africa are quite remarkable. If malarial vaccines are going to be tested in populations with a high frequency of these traits, particularly if they are attenuating vaccines, it will be extremely important to understand the genetic background of the populations being tested with respect to the frequency and potential interactions of these variants. And of course these interactions are also of major biological and evolutionary interest with respect to the distribution of the hemoglobin variants in the current global population.

The early data that has come from studies of the interaction of malaria with the more severe forms of thalassemia, notably sickle cell anemia and Hb E β thalassemia, are also of potential importance. It will be extremely important, for example, to repeat the susceptibility studies to *P. vivax* malaria of patients with Hb E β thalassemia in those parts of Asia where *P. vivax* malaria is presenting a major health problem and where Hb E β thalassemia is particularly common. Similarly, the observations of the apparent lack of susceptibility of patients with sickle cell disease to *P. falciparum* malaria provides important information about future design of programs for the better control and management of sickle cell anemia in African populations.

Finally, although some progress has been made the precise mechanisms of malaria protection by the hemoglobin variants and different forms of thalassemia is still uncertain. This is an important question because it is possible that if these mechanisms were better understood they might offer valuable clues to the development of completely different approaches to the control and management of malaria. It is interesting that the discovery that DARC is the receptor for *P. vivax* on red cells, which led to the discovery of the parasite antigen which interacts with this receptor, has led to the development of at least one promising target for vaccine production.^38^ It is very important to determine whether there are other lessons of this kind that remain to be learned from a better appreciation of the mechanisms of protection against malaria by other hemoglobin or related red cell polymorphisms.
